# Role of Matrilysins (MMP-7, MMP-26) and Stromelysins (MMP-3, MMP-10) in Diagnosing Cervical Cancer Patients

**DOI:** 10.3390/biomedicines13122910

**Published:** 2025-11-27

**Authors:** Ewa Gacuta, Michał Ławicki, Hanna Grabowska, Paweł Ławicki, Monika Kulesza, Aleksandra Kicman, Monika Zajkowska, Piotr Laudański, Sławomir Ławicki

**Affiliations:** 1 Department of Perinatology, University Clinical Hospital of Bialystok, 15-276 Bialystok, Poland; 2 Department of Population Medicine and Lifestyle Diseases Prevention, The Faculty of Medicine, Medical University of Bialystok, 15-269 Bialystok, Poland; mlawicki@icloud.com (M.Ł.); grabowska.hanna@icloud.com (H.G.); pawellawicki04@gmail.com (P.Ł.); monika.kulesza@sd.umb.edu.pl (M.K.); slawomir.lawicki@umb.edu.pl (S.Ł.); 3 Department of Aesthetic Medicine, Medical University of Bialystok, 15-089 Bialystok, Poland; olakicman@gmail.com; 4 Department of Neurodegeneration Diagnostics, Medical University of Bialystok, 15-269 Bialystok, Poland; monika.zajkowska@umb.edu.pl; 5 Department of Obstetrics, Gynecology and Gynecological Oncology, Medical University of Warsaw, 03-242 Warsaw, Poland; piotr.laudanski@wum.edu.pl

**Keywords:** cervical cancer, matrilysins, MMP-7, MMP-26, stromelysins, MMP-3, MMP-10, CA 125

## Abstract

**Background/Objectives**: Detection of cervical lesions is crucial for effective prevention and treatment. Therefore, the search for new diagnostic markers is extremely important. This study aimed to evaluate the concentration and diagnostic utility of selected matrix metalloproteinases (MMPs) as novel biomarkers. **Methods**: The study group consisted of 320 participants: 160 patients with cervical cancer (CC), 100 patients with cervical dysplasia (CD), and 60 healthy controls (HC). MMPs were determined by an ELISA (Enzyme-Linked Immunosorbent Assay), and CA 125 and SCC-Ag by a CMIA (chemiluminescent microparticle immunoassay). **Results**: Our study revealed significantly higher concentrations of MMP-7 and MMP-10 in the CC and CD groups compared to healthy controls. Interestingly, concentrations of these two parameters increased with the advancement of the disease. In the case of MMP-3, the highest concentrations were observed in the CD group, moderate concentrations in the group with diagnosed CC, and the lowest in the group of healthy women. Notably, the concentration of this parameter in the CC group decreased with increasing disease advancement. In the case of MMP-26, the highest concentrations, which increased with advancement, were observed in CC, moderate concentrations in HC, and the lowest in the group of women with CD. The highest diagnostic usefulness among all the parameters was shown for MMP-7 (sensitivity (SE): 96%; specificity (SP): 94.14%; positive predictive value (PPV): 92.26%; negative predictive value (NPV): 85.46%; area under the curve (AUC): 0.9878) and MMP-10 (SE: 90.25%; SP: 80.05%; PPV: 92.15%; NPV: 88.45%; AUC: 0.9404). **Conclusions**: All parameters presented significant differences between the concentrations obtained in the CC group and the CD group, which may indicate their usefulness not only in the diagnosis of cervical cancer, but also in the possible differentiation between benign and malignant lesions.

## 1. Introduction

Among the most prevalent cancers worldwide, cervical cancer (CC) ranks fourteenth in terms of incidence and is the fourth most common cancer among women [1,2]. Over 75% of CC cases are attributable to high-risk types of human papillomavirus (HPV), specifically types 16 and 18 [3,4]. There are over 130 known HPV types, 20 of which have been identified as associated with cancer. Persistent HPV infection is responsible for over 99% of all CC cases [1]. With this in mind, cervical cancer, which results mainly from persistent infection with sexually transmitted HPV, can be prevented through effective public health education. Vaccinations, which form a component of primary prevention, along with diagnostic tests such as cytology and HPV screenings, are regarded as the most effective methods for reducing the incidence of cervical cancer as well as the mortality rates caused by it [5,6].

Diagnostic procedures such as colposcopy and cytology are employed to monitor the progression of neoplastic changes [1]. High-risk lesions necessitate an individualized patient approach, taking into consideration the size, depth, and location of the lesions.

Despite the fact that most cervical cancer cases are detected at an early and therefore treatable stage, diagnostic and therapeutic challenges persist. This is partly due to variations in the number of screening tests conducted. At the same time, initiatives promoting vaccination are being increasingly intensified [7]. CEA, SCC-ag, and CA 19-9 are among the biomarkers measured using ELISA tests which are widely utilized in oncology, particularly for cervical cancer. However, none of these biomarkers are sufficiently specific for early detection of the disease [8]. Given the high mortality and recurrence rates associated with cervical cancer, there is a pressing need to identify new markers that can assess disease progression in women at risk.

The etiology of tumorigenesis is attributed to mutations in genes that impair cellular signaling pathways, potentially leading to uncontrolled tissue proliferation. Malignant cells forming a tumor interact with the vascularized stroma, which includes fibroblasts and myeloid cells. Within the tumor, angiogenesis is actively promoted, and the microenvironment exhibits processes similar to inflammatory responses observed during wound healing [1,9,10]. Carcinogenesis is influenced by invasive mobility, modulation of the microenvironment, plasticity, and colonization, as well as alterations in intercellular signaling and their communication with the extracellular matrix (ECM) [11]. Current research on the molecular mechanisms between the stroma and cancer cells suggests a role for extracellular proteases, such as matrix metalloproteinases (MMPs), as mediators in the reactions occurring within the tumor microenvironment during progression. While infiltrating stromal cells are considered the primary source of proteases, cancer cells from various tissues may also express these molecules [12,13].

MMPs are a group of zinc-dependent endopeptidases secreted by keratinocytes and fibroblasts. Within this enzyme family, we distinguish collagenases (MMP-1, MMP-8, and MMP-13), gelatinases (MMP-2 and MMP-9), stromelysins (MMP-3 and MMP-10), and membrane-type MMPs (MT1, 2, 3, 4, 5, and 6), as well as MMP-7, MMP-11, MMP-12, MMP-19, MMP-20, MMP-21, MMP-23, MMP-26, MMP-27, and MMP-28, categorized based on their structure [14]. They play a critical role in physiological processes such as tissue remodeling and organ development, and they also participate in the regulation of inflammatory processes [13,14]. MMPs may mediate cancer invasion and metastasis. Research indicates a correlation between excessive MMP expression and a more aggressive cancer phenotype, leading to poorer prognoses. Additionally, a relationship exists between MMP expression levels and tumor stages, suggesting a significant role for MMPs in the functioning of the microenvironment that initiates proliferation and angiogenesis in primary tumors and metastases [13,14,15,16]. MMP-7 (matrilysin) is crucial in regulating processes of aging, wound healing, growth, and bone remodeling, as well as in signaling pathways responsible for inflammatory responses and cell growth. It is expressed in tissues such as the endometrium, bronchi, ducts, glandular skin epithelium, urogenital tract, and gastrointestinal tract. MMP-7 also participates in the degradation of ECM substrates. Elevated levels of MMP-7 have been associated with various pathological conditions, and increased expression of MMP-7 has been demonstrated in human multi-organ tumors at the cellular level [14,17]. MMP-7 inhibits apoptosis in cancer cells, decreases cell adhesion, and induces angiogenesis. MMP-26 (matrilysin-2) is involved in the degradation of type IV collagen, fibronectin, fibrinogen, and vitronectin. MMP-3 and MMP-10, known as stromelysins 1 and 2, are enzymes that activate other metalloproteinases and initiate the degradation of collagen, elastin, fibronectin, gelatin, and laminin [14].

Due to the mortality associated with advanced stages of cancer and the challenges in early detection of cervical cancer, there is a need to search for new biochemical markers specific to the disease. Reports indicate a correlation between the activity of MMPs in cervical cancer and their influence on the tumor microenvironment [1,3,4]. In addition, this study examined the potential inclusion of MMP-7 and MMP-26, as well as MMP-3 and MMP-10, as prospective biomarkers indicative of disease progression, which would facilitate better identification of at-risk patients. The results were compared with the levels of CA125 and SCC-Ag obtained from the plasma of CC patients, and also in relation to the results obtained from a group of patients with cervical dysplasia (CD) and a control group (healthy subjects). Diagnostic criteria (sensitivity, specificity, positive and negative predictive values) and the receiver operating characteristic (ROC) curve for the parameters, studied both individually and in combinations, were established. Furthermore, a correlation among the analyzed biomarkers was determined. These data may be utilized to assess the utility of the investigated matrilysins (MMP-7, MMP-26) and stromelysins (MMP-3, MMP-10) in diagnosing patients with cervical cancer.

The present study is part of a broader research project aimed at evaluating the diagnostic potential of selected matrix metalloproteinases (MMPs) in various malignancies of epithelial origin. Each stage of this ongoing research focuses on a specific cancer type to provide detailed insights into the molecular mechanisms and clinical implications characteristic of that disease. In this paper, we concentrate on matrilysins (MMP-7, MMP-26) and stromelysins (MMP-3, MMP-10) in cervical cancer, which, despite belonging to the group of gynecological malignancies, differ substantially from other tumor types previously analyzed in terms of etiological factors, hormonal background, and the tumor microenvironment. Such an approach ensures thematic and biological coherence within each manuscript, while collectively contributing to a comprehensive understanding of MMP dysregulation across cancer types.

## 2. Materials and Methods

### 2.1. Materials

This study consisted of 320 participants divided into 3 groups: patients with cervical cancer (CC) (stages I–II and stages III–IV), patients with cervical dysplasia (CD), and a healthy control group (HG). A total of 160 patients with cervical cancer underwent diagnosis and subsequent surgical treatment at the University Clinical Hospital in Bialystok. The cervical dysplasia group, which consisted of 100 patients, was diagnosed and kept under observation. The control group consisted of 60 healthy women, without any abnormalities within their uterine cervixes. Characteristics of the studied groups as well as exclusion and inclusion criteria are presented in Table 1.

We qualified patients with cervical cancer or cervical dysplasia on the basis of gynecological examinations, followed by confirmatory examinations by an oncologist on the basis of imaging studies (USG/magnetic resonance imaging) and laboratory tests. Cervical cancer (CC) cases were staged according to the FIGO (International Federation of Gynecology and Obstetrics) classification, with patients divided into stages I–II and III–IV. Cervical dysplasia (CD) cases were assessed histopathologically and classified using the CIN (Cervical Intraepithelial Neoplasia) system, with cases included as “benign” corresponding to CIN 1–2.

The healthy women in the control group were volunteers who were first assessed and deemed eligible for participation by a family doctor, and then underwent detailed imaging and laboratory evaluations conducted by a gynecologist from the University Clinical Hospital in Bialystok and participants of the Bialystok PLUS cohort study. After the women underwent abdominal or intravaginal ultrasound/magnetic resonances, the gynecologist subsequently determined the possibility of the women’s inclusions in the study.

### 2.2. Methods

Blood samples were collected into S-Monovette tubes and processed according to Good Laboratory Practice and Standard Operating Procedures. Within 30 min of collection, samples were centrifuged (10 min, RCF = 10,000). After centrifugation, plasma was separated from the red blood cell fraction and aliquoted into sterile 0.5 mL Eppendorf tubes. All tubes were labeled and stored in cardboard racks at −85 °C. Sample preparation was performed at a controlled room temperature of 21 °C.

We measured plasma matrilysin (MMP-7, MMP-26) and stromelysin (MMP-3, MMP-10) concentrations with the use of the immuno-enzymatic ELISA method (Quantikine ELISA Human, R&D Systems Inc., Minneapolis, MN, USA). The assays were performed according to the manufacturer’s instructions provided with the kits, using double sample determinations for the standard curve and tested samples. For measurement of CA 125 and SCC-Ag levels, we used a chemiluminescent microparticle immunoassay (CMIA) (Abbott, Chicago, IL, USA) according to the manufacturer’s protocols.

### 2.3. Statistical Analysis

Statistical analysis was performed using the statistical program PQStat Software (v.1.8.4.162, Poznan, Poland); graphical processing was performed using GraphPad Prism Software (v.9.1.1 (225), San Diego, CA, USA). After the evaluation of the normality of the distribution for the tested parameters with the D’Agostino and Pearson omnibus normality test, which revealed significant deviations from the normal distribution, we performed statistical analysis using nonparametric tests. To assess statistical differences between three independent groups, we used the Kruskal–Wallis statistical test.

For the evaluation of the diagnostic features of the tested parameters, i.e., diagnostic sensitivity (SE), diagnostic specificity (SP), positive predictive value (PPV), negative predictive value (NPV), and diagnostic power, our analysis was performed on the basis of the area under the ROC curve (AUC) and the optimal cut-off points determined by the method with the 95th percentile of the healthy control group, which were, respectively, MMP-7—1.608 ng/mL, MMP-26—18.67 ng/mL, MMP-3—9.122 ng/mL, MMP-10—65.02 ng/mL, CA125—16.19 U/mL, and SCC-Ag—0.9778 ng/mL. A *p*-value of less than 0.05 was considered statistically significant.

## 3. Results

Plasma concentrations of the studied parameters in patients with cervical cancer (CC) divided into stages I–II and III–IV, cervical dysplasia (CD), and control patients are shown in Table 2. What is important is that we observed clear trends in the distribution of all studied parameters across patient groups. MMP-7 and MMP-10 showed the most pronounced increases in cervical cancer patients, with median concentrations rising progressively from cervical dysplasia to early-stage (I–II) and advanced-stage (III–IV) cancer. This pattern indicates a potential relationship between these markers and disease progression. MMP-26 also displayed elevated concentrations in cervical cancer patients compared to controls, although the increase was less pronounced. In contrast, MMP-3 levels were significantly lower in cancer patients relative to the dysplasia group, suggesting a distinct behavior compared to the other metalloproteinases. Statistical analysis showed that patients with cervical cancer had significantly higher levels of MMP-7 in stages I–II (median 491.60 ng/mL) and III–IV (566.90 ng/mL), MMP-10 in stages I–II (median 1586 ng/mL) and stages III–IV (2042 ng/mL), and MMP-26 in stages I–II (median 8.77 ng/mL) and stages III–IV (9.31 ng/mL) compared to patients with cervical dysplasia (CD) (median: MMP-7: 126.60 ng/mL; MMP-10: 484.30 ng/mL; MMP-26: 1.51 ng/mL). MMP-3, on the other hand, showed significantly lower values in CC patients in stages I–II (median 11.01 ng/mL) and stages III–IV (9.97 ng/mL) compared to the median MMP-3 value obtained in the CD group (35.34 ng/mL) (*p* < 0.0001 for each parameter).

The obtained median plasma concentrations in CC patients were compared with the results obtained in the control group. When comparing cancer patients with healthy controls, all MMPs demonstrated significant differences (*p* < 0.0001), highlighting their potential utility as diagnostic biomarkers. Among standard markers, CA125 and SCC-Ag reached the lowest values for sensitivity, specificity, PPV, and NPV, whereas MMP-7 and MMP-10 consistently showed the highest diagnostic performance. We obtained significantly higher values of MMP-3 in CC patients in stages I–II (median 11.01 ng/mL) and stages III–IV (9.97 ng/mL), MMP-7 in stages I–II (median 491.60 ng/mL) and stages III–IV (566.90 ng/mL), MMP-10 in stages I–II (median 1586 ng/mL) and stages III–IV (2042 ng/mL), and MMP-26 in stages I–II (median 8.77 ng/mL) and stages III–IV (9.31 ng/mL) compared to the results obtained in the healthy control group (median: MMP-3: 6.94 ng/mL; MMP-7: 0.88 ng/mL; MMP-10: 44.65 ng/mL; MMP-26: 7.84 ng/mL) (*p* < 0.0001 for each parameter).

Table 3 contains the diagnostic criteria—diagnostic sensitivity (SE), diagnostic specificity (SP), and positive and negative predictive values (PPV and NPV)—for cervical cancer patients.

Standard biomarkers CA125 (59%) and SCC-Ag (56%) reached the lowest values of SE. Furthermore, all of the tested MMPs surpassed them greatly, at 61% (MMP-26), 66.45% (MMP-3), 90.25% (MMP-10), and the highest, 96%, for MMP-7.

The outcome of SP measurement showed that CA125 (55.27%), and SCC-Ag (51.03%) also reached the lowest scores and were surpassed by every MMP (range: 56.05–94.14%). The highest SP value was reached by MMP-7 (94.14%).

The highest PPV values, 92.26% and 92.15%, were respectively obtained by MMP-7 and MMP-10. The lowest values were obtained by CA125 and SCC-Ag, which reached 42% and 41%, respectively.

MMP-7 and MMP-10 exhibited also the highest NPV values (MMP-7 at 85.46% and MMP-10 at 88.45%). In contrast, CA125 and SCC-Ag demonstrated almost the lowest NPV values, at 53.55% and 55.56%, respectively. MMP-7 and MMP-10 not only differentiate cervical cancer from dysplasia and healthy controls but also reflect disease stage, supporting their potential clinical application as sensitive and specific serum biomarkers. Furthermore, the distribution patterns and statistical significance observed across all parameters underscore the robustness and reliability of the findings.

**Figure 1:**
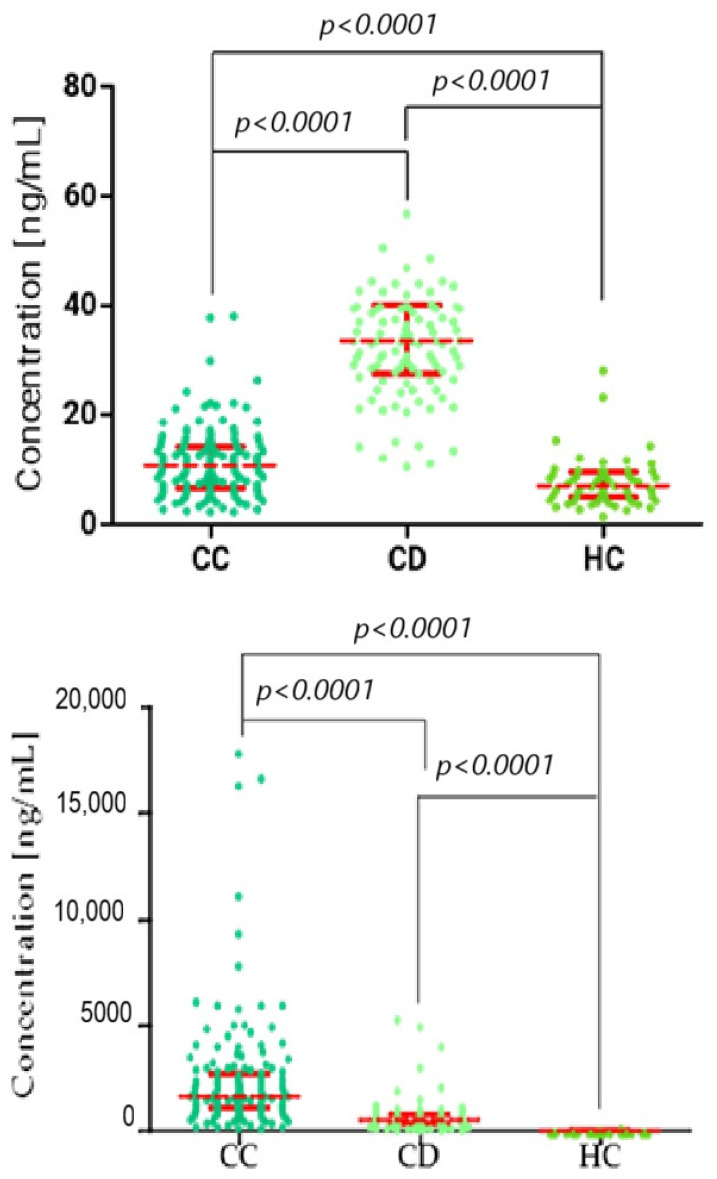
Kruskal–Wallis/Dunn’s Multiple Comparison Test chart, showing concentration distributions for stromelysins (top: MMP-3; and bottom: MMP-10) in the cervical cancer (CC), cervical dysplasia (CD), and healthy control (HC) groups. Statistically significant correlation between groups. Red bars represent the median values, and the error bars indicate the interquartile range (IQR) of the data.

**Figure 2:**
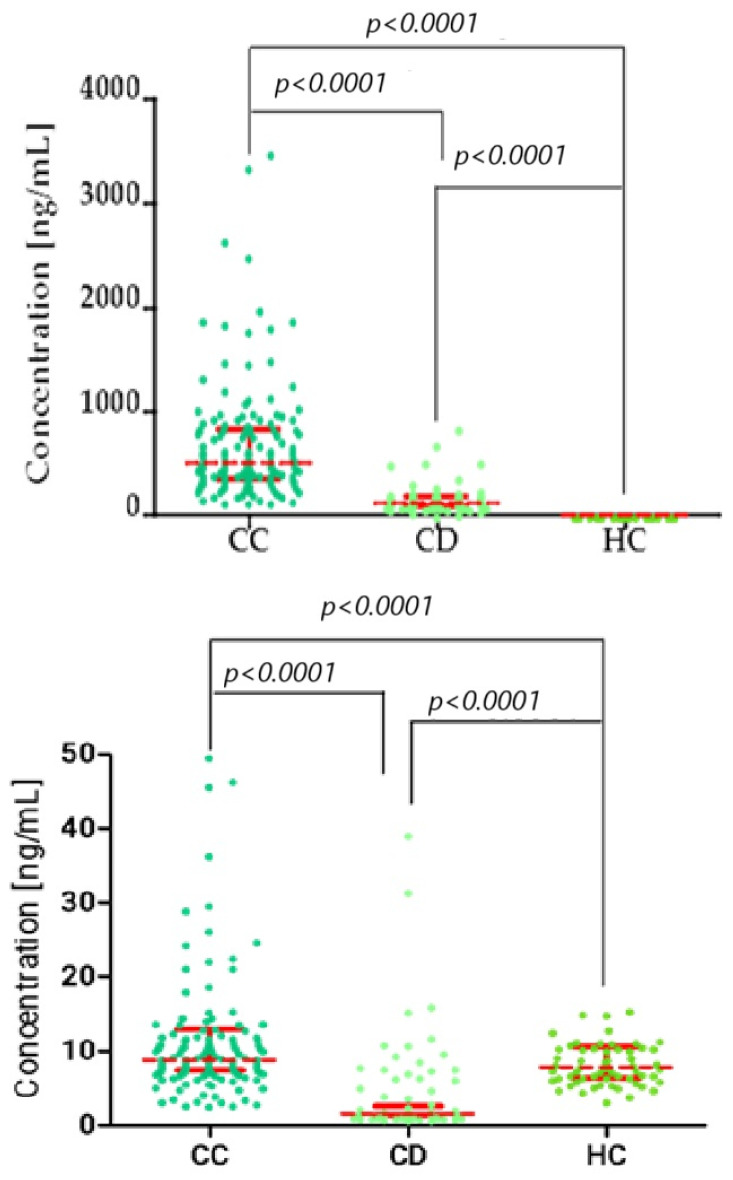
Kruskal–Wallis/Dunn’s Multiple Comparison Test chart, showing concentration distributions for matrilysins (top: MMP-7; bottom: MMP-26) in the cervical cancer (CC), cervical dysplasia (CD), and healthy control (HC) groups. Statistically significant correlation between groups. Red bars represent the median values, and the error bars indicate the interquartile range (IQR) of the data.

**Figure 3:**
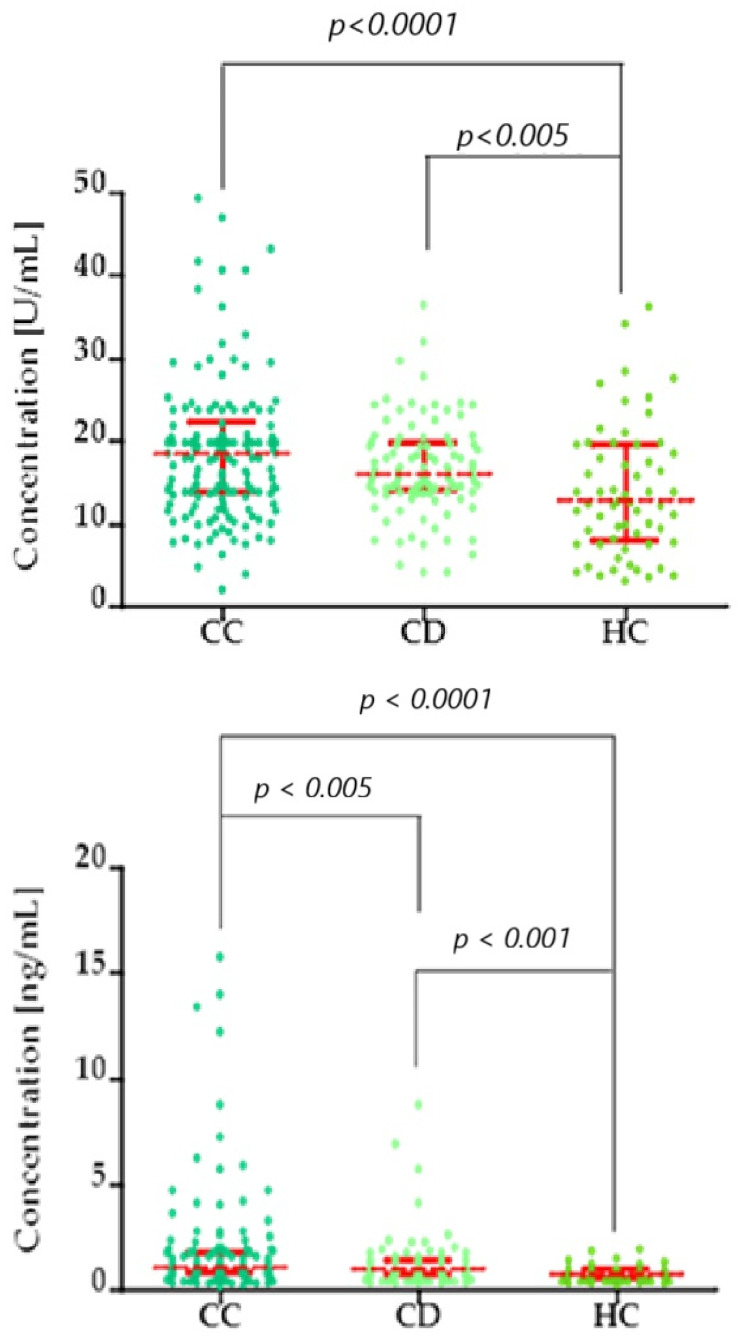
Kruskal–Wallis/Dunn’s Multiple Comparison Test chart, showing concentration distributions (top: CA125; bottom: SCC-Ag) in the cervical cancer (CC), cervical dysplasia (CD), and healthy control (HC) groups. Red bars represent the median values, and the error bars indicate the interquartile range (IQR) of the data.

To assess statistical differences between two independent groups, we used the Kruskal–Wallis/Dunn’s Multiple Comparison Test, comparing multiple groups. Figure 1, Figure 2 and Figure 3 present the statistical distribution of stromelysins (MMP-3 and MMP-10), matrilysins (MMP-7, MMP-26), and comparative markers (CA125, SCC-Ag) in cervical cancer patients (CC), cervical dysplasia patients (CD), and the healthy control (HC) group. Furthermore, correlation between the tested groups is presented with statistical significance.

**Figure 4:**
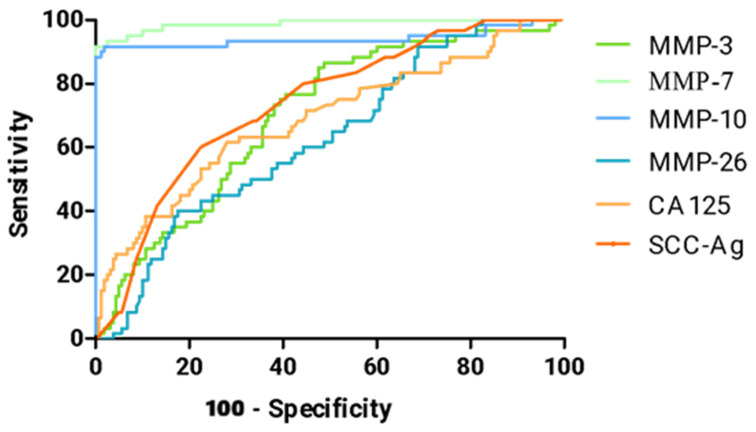
ROC curve for tested parameters in cervical cancer group.

The ROC curve represents the relationship between SE and SP for the parameters being studied, while the AUC value reflects the potential clinical utility of a parameter as a tumor marker and helps to assess its diagnostic power. The detailed parameters from the ROC curve analysis are provided in Table 4 and Figure 4. For ROC analyses, only cervical cancer patients were included as the positive group, and healthy women served as the negative (control) group.

Table 4 shows a summary of all AUC values for matrilysins (MMP-7, MMP-26), stromelysins (MMP-3, MMP-10), CA 125, and SCC-Ag in the cervical cancer patient group. All values were significantly higher compared to AUC = 0.5 (*p*-value in red in the table).

The highest AUC value in cervical cancer patients was obtained by MMP-7 (0.9878), which greatly surpasses the results of standard comparative markers CA 125 (0.6870) and SCC-Ag (0.7352). The second MMP that outperforms the standard comparative markers is MMP-10, which reached AUC = 0.9404.

These results indicate that MMP-7 and MMP-10 not only differentiate cervical cancer from dysplasia and healthy controls but also reflect disease stage, supporting their potential clinical application as sensitive and specific serum biomarkers. Furthermore, the distribution patterns and statistical significance observed across all parameters underscore the robustness and reliability of the findings.

## 4. Discussion

Detection of cervical lesions, both benign and malignant, is crucial for effective prevention and treatment of cervical cancer. In the current diagnostic system, based mainly on screening tests such as cytology and HPV tests, there are limitations in detecting some lesions, which can lead to a delay in diagnosis. Therefore, the search for new diagnostic markers, especially ones which are useful in everyday practice, is extremely important. Biomarkers can improve the sensitivity and specificity of detecting precancerous lesions and early stages of cancer, which in turn will allow for earlier intervention and increase the chance of full recovery. The development of these diagnostic tools is a key step towards more effective prevention of and therapy for cervical diseases. It has been proven that the enhanced activity of MMPs is strongly linked to a number of tumors, including gynecological cancers [18,19,20,21]. The current findings should be interpreted as part of a consistent research series investigating the diagnostic significance of matrix metalloproteinases in different malignancies. The present study represents a subsequent phase of this work, extending our earlier observations to cervical cancer. Given the large number of MMPs and the extensive dataset obtained, it was not feasible to present all results within a single comprehensive manuscript, as this would have considerably limited the clarity and interpretability of the data. Therefore, we decided to publish these results as a series of related studies, each devoted to one specific tumor type. This strategy enables focused discussion of disease-specific mechanisms, while preserving methodological consistency and comparability across the series.

In the present study, we investigated the usefulness of selected metalloproteinases, MMP-3, MMP-7, MMP-10, and MMP-26, as well as CA 125 and SCC-Ag (commonly used tumor markers), in patients with CC (in particular cancer stage subgroups—stages I + II, and III + IV) and patients with dysplasia in comparison to healthy women. Our results showed significantly higher concentrations of MMP-7 and MMP-10 in the CC and CD groups compared to healthy controls. Interestingly, the concentrations of those two parameters increased with the advancement of the disease, as moderate concentrations were observed in benign lesions, higher in less advanced stages (I–II) of cancer, and the highest in highly advanced stages (III–IV). In the case of MMP-3, the highest concentrations were observed in the CD group, moderate concentrations in the group with diagnosed CC, and the lowest in the group of healthy women. Importantly, the concentration of this parameter in the CC group decreased with increasing disease advancement. In the case of MMP-26, the highest concentrations and those increasing with advancement were observed in CC, moderate concentrations in the healthy group (HG), and the lowest in the group of women with diagnosed dysplasia. Concentrations of comparative markers (CA 125 and SCC-Ag) performed in a manner similar to MMP-7 and MMP-10. However, the observations indicate less pronounced dynamics of CA 125 and SCC-Ag changes, yet these are still significant, suggesting the beneficial influence of the tested parameters, especially MMP-7 and MMP-10.

In the work of Wu et al. [22] concerning MMP-7, the authors described identical relationships as observed in our study, in that both mRNA expression and MMP-7 protein level in cervical cancer tissues were the lowest in healthy cervical tissues, were higher in the group with no metastases present, and were the highest in the group of high-malignancy tissues with lymph metastases present. The authors also emphasized that due to the observed dynamics, this parameter can be used as a marker in the assessment of the invasive and metastatic potential of cervical cancer. Additionally, in the work of Guo et al. [23], tissue expression of MMP-7 was significantly higher in patients with CC compared to healthy tissues, and was more frequently observed in patients with metastases to pelvic lymph nodes, which confirms previously described reports and correlation of MMP-7 with invasive potential. Similar observations were also noted by other authors, who additionally proved that silencing of the MMP-7 gene significantly decreased CC proliferation, migration, and invasion [24], and also showed that this gene was associated with decreased overall survival [25]. Therefore, this confirms our reports using a test material other than blood, making MMP-7 a good candidate for a biomarker of cervical cancer. However, our work is the only one also assessing MMP-7 concentration in patients with benign lesions, which is an innovative element, and the result of the assessment of differences in concentrations between groups taking into account benign lesions provides new information. There are also available works in the literature attributing a high risk of CC to one of the MMP7 gene polymorphisms (rs11568818), which is worth special attention, but these data need to be confirmed on a larger scale, as they concern only Asian populations [26,27].

In the case of MMP-10, there are no available studies describing the diagnostic usefulness of this MMP in cervical cancer; therefore, our study is innovative. However, there are available results of the authors’ work indicating the association of high MMP-10 concentration with HPV infection, which is indirectly related to the later development of CC [28]. There is also available work by Li et al. [29] describing studies on a new therapeutic target (EFEMP2) in CC on highly invasive cell lines (Ca Ski), where knockdown of the proposed therapeutic target gene was simultaneously associated with a decrease in MMP-10 expression. This may also indirectly confirm the theory that high expression and concentrations of MMP-10 may be associated with and correspond to, just like MMP-7, an increase in cancer progression. Similar results with Ca Ski lines were observed in the work of Liao et al. [30]. A comparison of MMP-10 expression in benign lesions and CC was presented in the work of Zhang et al. [31], where mainly low reactivity for MMP-10 in benign lesions and high immunoreactivity in most of the cancer samples was demonstrated. The described relationships in tissue expression are consistent with our results for MMP-10 concentrations in blood plasma.

In the work of Argüello-Ramírez et al. [32], the authors compared the secretion of MMPs to the conditioned medium in the case of benign and CC lesions; they showed that high concentrations of MMP-3 are associated with neoplastic changes. However, they did not show an increased concentration of MMP-3 in the case of benign lesions. These differences, specifically in the methodology and type of samples tested, may be related to the small number of samples included in the described study. However, the authors noticed in the course of their work that MMP-3 secretion can be a marker of poor CC prognosis. Interestingly, in the work of Shao et al. [20], the authors proved that the expression and concentration of MMP-3 is also significantly higher in patients with CC compared to healthy women. Unfortunately, the work did not include an analysis of women with diagnosed benign cervical lesions. The authors also proved a downward trend in MMP-3 after treatment, which is an important element confirming the pro-neoplastic nature of this MMP. High concentrations of this metalloproteinase were also associated with low overall survival rates. The authors also decided to investigate MMP3 knockdown and proved that it could lead to enhanced apoptosis levels in HT-3 cell lines [20]. In the work of Okusha et al. [33] the authors also confirmed that MMP-3 expression correlated with the prognosis of CC and was associated with lower overall survival. Moreover, as with MMP-10, work by Li et al. [29] described studies on a new therapeutic target (EFEMP2) in CC on highly invasive cell lines (Ca Ski), where knockdown of the proposed therapeutic target gene was simultaneously associated with a decrease in MMP-3 expression, which may also indirectly confirm the theory that high expression and concentrations of MMP-3 may be associated with cancer progression. Similar reports have also been published by other authors [30,34]. This is why we suggest that MMP3 has a potential application in the early diagnosis, screening, and therapy of CC.

Our data on MMP-26 in cervical cancer are the first such reports. Unfortunately, there are no available papers describing the diagnostic utility or even tissue/gene expression of MMP-26 in cervical cancer; therefore, we are not able to compare our results with those of other authors. Our reports in this case should be considered as pioneering studies that require further confirmation using other techniques and test materials. However, there are studies available on this MMP in other gynecological cancers; e.g., in the case of endometrial or ovarian cancer, higher concentrations of MMP-26 were demonstrated when compared to healthy women [18,35].

The next stage of our work was to assess the diagnostic usefulness of the parameters studied. As was possible to establish, the highest diagnostic usefulness among all the parameters was for MMP-7 (SE: 96%; SP: 94.14%; PPV: 92.26%; NPV: 85.46%; AUC: 0.9878) and MMP-10 (SE: 90.25%; SP: 80.05%; PPV: 92.15%; NPV: 88.45%; AUC: 0.9404). The remaining parameters studied showed slightly higher or comparable usefulness compared to routine markers. Unfortunately, we could not compare our data with the findings of other authors. The majority of results in the available literature concern tissue expression or different evaluations of the parameters, as described earlier. The only works that were able to assess AUC were the works of Shao et al. [20] and Guo et al. [22] (based on the tissue expression). In the first paper, the authors determined the AUC for serum MMP-3 at the level of 0.9196, which was significantly higher than the value we obtained. In the second paper, the AUC for MMP-7 was determined at the level of 0.707, which also indicates the high usefulness of this parameter, but it is a much lower value than the one obtained in our assignment. These differences may result from the type of studies conducted (concentration vs. expression).

However, we believe that this article thoroughly demonstrates the potential screening utility of MMPs, especially MMP-7 and MMP-10, in diagnosing women with CC. What is worth mentioning is that all parameters showed significant differences between the concentrations obtained in the CC group and the CD group, which may indicate their usefulness not only in the diagnosis of CC but also in the possible differentiation between benign and malignant lesions at the diagnostic stage, before performing histopathological tests.

The findings of the present study suggest that selected MMPs, particularly MMP-7 and MMP-10, could be integrated into current diagnostic algorithms for cervical lesions as adjunctive plasma biomarkers. Given their high sensitivity and specificity, these markers could complement existing screening methods such as cytology and HPV testing, potentially improving early detection of both benign and malignant lesions. In practice, measuring MMP-7 and MMP-10 concentrations in patients presenting for routine screening or follow-up could help prioritize individuals for further histopathological evaluation, thereby reducing the risk of missed or delayed diagnoses. Furthermore, the observed ability of these markers to differentiate between benign dysplasia and cervical cancer indicates that they could support risk stratification and inform personalized patient management strategies, enhancing the clinical utility of current diagnostic workflows.

Despite the promising results, several limitations of this study should be acknowledged. Although the AUC values for MMP-7 and MMP-10 were very high, suggesting excellent diagnostic performance, we acknowledge that these results may overestimate the true diagnostic accuracy. Independent validation in larger and more diverse cohorts is needed to confirm these findings. Reanalysis of our data confirmed the original results. The sample size was relatively small, which may limit the generalizability of the findings. Additionally, the study population was relatively homogeneous in terms of demographic and clinical characteristics, which could influence the observed marker concentrations. Future studies with larger and more diverse cohorts are needed to validate these findings and to confirm the diagnostic utility of MMP-3, MMP-7, MMP-10, and MMP-26 across different populations and clinical settings, as this work is an introduction to further development of the usefulness of the described parameters and confirmation of these assumptions on a larger scale.

## 5. Conclusions

In conclusion, this study highlights the importance of early detection of cervical lesions, which in the future may influence more effective prevention and treatment of cervical cancer. In our study we tried to assess the potential of MMP-3, MMP-7, MMP-10, and MMP-26, which showed significant differences in concentrations between the groups of healthy women, dysplasia patients, and cancer patients, and also demonstrated the ability to distinguish benign from malignant lesions. MMP-7 and MMP-10 had the greatest diagnostic value, suggesting their potential as screening and prognostic markers. Our results showed that the study of these biomarkers can support early detection of cervical cancer and assessment of the disease stage, which is important for improving the effectiveness of treatment and patient prognosis. We believe that our results are an important step towards the development of more precise diagnostic tools and therapies in gynecological oncology.

## Figures and Tables

**Table 1:** Characteristics of examined groups: cervical cancer (CC) group of patients, cervical dysplasia (CD) group of patients, and healthy control group (HG).

Studied Group	Group Size		Median Age (Min–Max)	Exclusion Criteria	Inclusion Criteria
cervical cancer group (CC)	stages I–II	120	46 (25–61)	• Age < 18 years • BMI > 30 kg/m^2^ Pregnancy or postpartum period • Active infection or inflammatory disease at the time of sampling • Autoimmune or chronic systemic disease affecting inflammatory markers • Previous cervical surgery or oncological treatment prior to enrollment • Hemolyzed or poor-quality blood samples • Lack of consent to participate in the study	• Age ≥ 18 years • Confirmed diagnosis of cervical cancer
	stages III–IV	40			
cervical dysplasia group (CD)	100		44 (23–60)		• Age ≥ 18 years • Confirmed benign cervical lesion
healthy control group (HG)	60		42 (22–61)		• Age ≥ 18 years • No history or current diagnosis of any malignancy or cervical pathology

**Table 2:** Plasma levels of tested MMPs, CA 125, and SCC-Ag in cervical cancer patients, cervical dysplasia patients, and healthy control group.

Tested Parameters	Cervical Cancer (CC) Median Range		Cervical Dysplasia (CD) Median Range	Healthy Control Group (HG) Median Range
MMP-3 (ng/mL)	Total group	10.63 2.62–38.55	35.34 20.96–57.26	6.94 1.81–28.59
	Stages I–II	11.01 2.62–38.55		
	Stages III–IV	9.97 2.86–24.65		
MMP-7 (ng/mL)	Total group	505.20 132.70–3474.00	126.60 6.25–237.10	0.88 0.04–5.23
	Stages I–II	491.60 132.70–2639.00		
	Stages III–IV	566.90 160.10–3474.00		
MMP-10 (ng/mL)	Total group	1539.00 266.90–17,898.00	484.30 131.80–5337.00	44.65 12.30–249.50
	Stages I–II	1586.00 272.20–17,898.00		
	Stages III–IV	2042.00 266.90–16,751.00		
MMP-26 (ng/mL)	Total group	8.77 2.78–116.30	1.51 0.83–50.26	7.84 3.42–15.60
	Stages I–II	8.77 2.78–115.60		
	Stages III–IV	9.31 2.91–116.30		
CA125 (U/mL)	Total group	18.56 2.53–259.10	16.10 4.70–59.90	12.95 3.50–36.60
	Stages I–II	16.70 2.53–77.41		
	Stages III–IV	24.85 5.30–259.10		
SCC-Ag (ng/mL)	Total group	1.10 0.30–78.56	1.00 0.50–8.90	0.80 0.40–2.10
	Stages I–II	1.00 0.30–14.10		
	Stages III–IV	2.10 0.50–78.56		

**Table 3:** Diagnostic criteria (SE, SP, PPV, and NPV) for the studied parameters in the group of patients with CC.

Diagnostic Criteria (%)	MMP-3	MMP-7	MMP-10	MMP-26	CA125	SCC-Ag
SE	66.45	96.00	90.25	61.00	59.00	56.00
SP	72.56	94.14	80.05	56.05	55.27	51.03
PPV	65.22	92.26	92.15	45.45	42.00	41.00
NPV	63.74	85.46	88.45	52.04	53.55	55.56

**Table 4:** Characteristics of ROC curve for tested parameters in patients with cervical cancer.

Tested Parameter	AUC	SE(AUC)	95% C.I.	*p* (AUC = 0.5)
MMP-3	0.6992	0.03768	0.6253–0.7730	<0.0001
MMP-7	0.9878	0.00735	0.9734–1.0020	<0.0001
MMP-10	0.9404	0.02678	0.8879–0.9929	<0.0001
MMP-26	0.6282	0.04022	0.5494–0.7071	0.0034
CA125	0.6870	0.04206	0.6045–0.7694	<0.0001
SCC-Ag	0.7352	0.03621	0.6642–0.8061	<0.0001

Red color—statistically significant.

## Data Availability

The data presented in this study are available on request from the corresponding author. Key data are stated in the text.

## Abbreviations

CC	cervical cancer
HPV	human papillomavirus
ECM	extracellular matrix
MMPs	metalloproteinases
CD	cervical dysplasia
CMIA	chemiluminescent microparticle immunoassay
SE	diagnostic sensitivity
SP	diagnostic specificity
PPV	positive predictive value
NPV	negative predictive value
HG	healthy control group
